# In vivo blockade of acetylcholinesterase increases intraovarian acetylcholine and enhances follicular development and fertility in the rat

**DOI:** 10.1038/srep30129

**Published:** 2016-07-21

**Authors:** Javier Urra, Jan Blohberger, Michelle Tiszavari, Artur Mayerhofer, Hernan E. Lara

**Affiliations:** 1 Laboratory of Neurobiochemistry, Faculty of Chemistry and Pharmaceutical Sciences, Universidad de Chile, 8380492 Independencia, Santiago, Chile; 2 BMC, Cell Biology, Anatomy III, Ludwig-Maximilian-University (LMU), 82152 Planegg, Germany

## Abstract

Growth and differentiation of ovarian follicles are regulated by systemic and local factors, which may include acetylcholine (ACh). Granulosa cells (GCs) of growing follicles and luteal cells produce ACh and in cultured GCs it exerts trophic actions via muscarinic receptors. However, such actions were not studied *in vivo*. After having established that rat ovarian GCs and luteal cells express the ACh-metabolizing enzyme ACh esterase (AChE), we examined the consequences of local application of an AChE inhibitor, huperzine A (HupA), by osmotic minipump delivery into the ovarian bursa of hemiovariectomized rats. Saline was used in the control group. Local delivery of HupA for 4 weeks increased ovarian ACh content. Estrus cyclicity was not changed indicating a locally restricted range of HupA action. The number of primordial and primary follicles was unaffected, but small secondary follicles significantly increased in the HupA group. Furthermore, a significant increase in the number of corpora lutea suggested increased ovulatory events. In support, as shown upon mating, HupA-treated females had significantly increased implantation sites and more pups. Thus the data are in support of a trophic role of ACh in follicular development and ovulation and point to an important role of ACh in female fertility.

The crucial functions of the ovary are regulated by multiple systemic and local signaling factors. Locally produced neurotransmitters and neurotransmitters released from autonomic nerves have been demonstrated to be involved in follicular development, atresia and ovulation^1^,^2^,^3^. Norepinephrine (NE), the main sympathetic neurotransmitter, for example, participates in the control of follicular development and steroid secretion^1^,^3^. In addition, chronic sympathetic stimulation, e.g. induced by cold stress, has been demonstrated to disrupt ovarian follicular development and to produce a polycystic ovary phenotype^4^,^5^.

Ovarian NE appears to have neuronal origin, but in contrast acetylcholine (ACh), the prototype parasympathetic neurotransmitter, is synthesized within the ovary by non-neuronal cells, namely granulosa cells (GCs). They represent the main cellular components of the ovarian follicle, a structure which is devoid of any nerve fibers^6^. GCs also express muscarinic receptors for ACh. Thus an ovarian cholinergic system has been postulated, which is part of a body-wide system of non-neuronal ACh production and local ACh actions. The physiological significance of this system in the ovary and its functions is not clear yet^7^. Previous studies, however, identified trophic, growth-promoting influences of ACh on GCs via muscarinic receptors in bovine and human ovary-derived ovarian cells. The documented consequences include increases in intracellular Ca^2+^ levels, activation of ion channels and disruption of gap junctional communication^8^,^9^.

In the brain the actions of ACh are strictly regulated by enzymatic breakdown of ACh. Similar mechanisms of breakdown of ACh and thus restriction of the availability of ACh to ovarian cells may be assumed. Two enzymes can be involved, butyrylcholinesterase (BuChE) and acetylcholinesterase (AChE). BuChE was reported in human follicular fluid^10^,^11^. Recently, functional AChE was described there as well^9^. The same study also revealed that at least three AChE isoforms, derived from alternative splicing, are present in the human and primate ovary and in granulosa cells. One of the AChE forms, AChE-R, furthermore participates in the control death of follicular cells and induced necroptosis, i.e. a form of cell death that had not been described previously in the ovary.

The presence of ovarian AChE allows pharmacological modulation of its function. In a previous study^9^, the specific AChE inhibitor huperzine A (HupA)^12^ was successfully used in cultures of human GCs and this approach further supported the trophic actions of elevated ACh. HupA has several advantages over other blockers of AChE (e.g. tacrine, donepezil and rivastigmine), including a longer duration of the AChE inhibitory action. HupA is also more potent AChE inhibitor compared to tacrine, rivastigmine and galantamine and has the least anti-butyrylcholinesterase activity among tested inhibitors (see^12^). In the present work we wanted to extend our investigation from the cellular to the systemic levels and turned to the rat model. To this end, and after having established that AChE is present in the follicular compartment of rats, we examined the consequences of a 4-week intrabursal local delivery of HupA on ovary function, follicular growth and fertility in the rat.

## Results

### Cholinesterases in the rat ovary and granulosa cells

**Figure 1:**
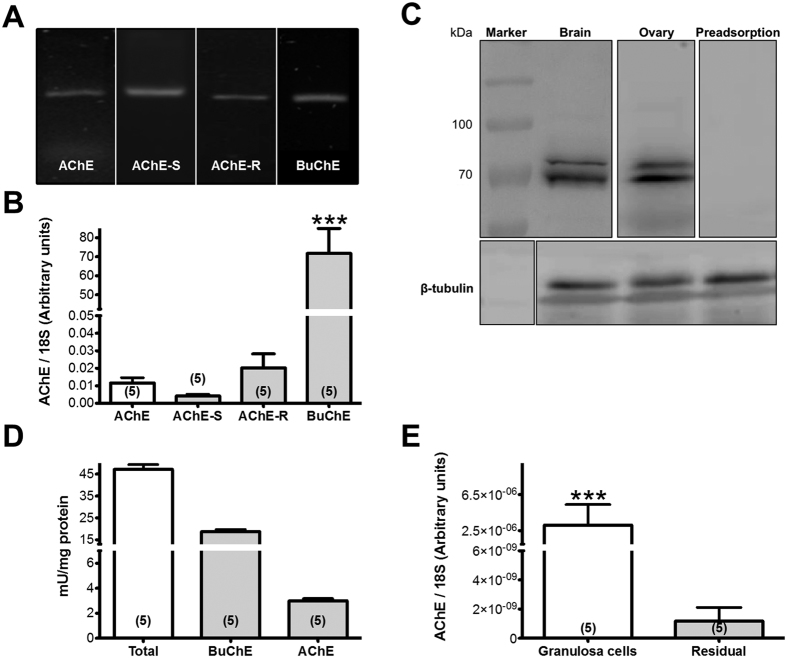
Detection of AChE in the rat ovary. (**A**) Results of RT-PCR experiments (ethidum bromide stained gel; different gels under same experimental conditions). The bands correspond to AChE, isoforms of AChE and BuChE. (**B**) qPCR data in bars correspond to means + S.E.M. of n = 5 independent samples (***P < 0.001). (**C**) Western blot analysis of AChE and β-tubuline control in the ovary and brain (different blots under same experimental conditions). Note two bands, which may be splice variants. Preadsorption of ovarian protein abolished the specific staining of the protein bands. (**D**) Results of cholinesterase activity measurements determined by Ellman assay. Results are the mean + S.E.M. of n = 5 independent measurements. (**E**) Distribution of AChE between granulosa/luteal cells and theca/interstitial cells determined by qPCR. Results are the mean + S.E.M. of n = 5 samples (*** *P* < 0.001).

Two types of cholinesterases were detected in the rat ovary. Reverse transcription PCR (RT-PCR) studies detected AChE and two of its isoforms AChE-S and -R, as well as BuChE (Fig. 1A). Quantification of the relative amount of mRNA by real time PCR (qPCR) showed that BuChE dominates and that AChE-S and -R represent the majority of AChE (Fig. 1B). Ovarian expression of the corresponding proteins of AChE and BuChE were confirmed by western blot analysis and rat brain was used as positive control (Fig. 1C). Cholinesterase activity was determined and the minor component was identified as active AChE, while BuChE activity dominates in total ovary homogenates (Fig. 1D). To estimate the relative distribution and abundance of AChE mRNA we isolated a crude fraction of granulosa-luteal cells and one of the residual ovary fraction (Fig. 1E). We found a preferential abundance in the granulosa-luteal cells fraction.

**Figure 2:**
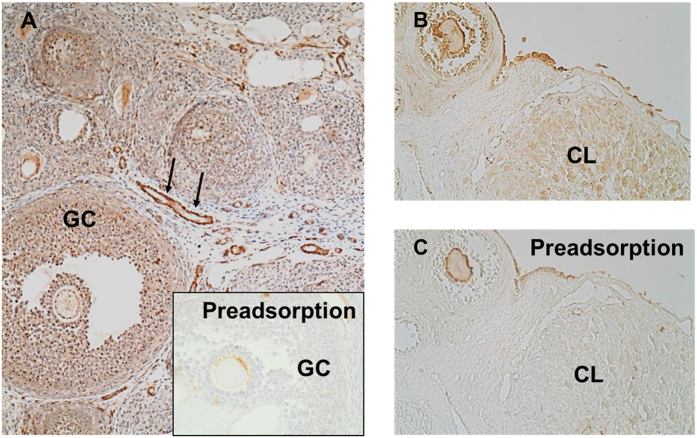
Immunohistochemical detection of AChE in rat ovary. (**A**) GCs of preantral and antral follicles are positive for AChE in an immunohistochemical staining. AChE was also associated with blood vessels (arrows). Preadsorption of the antibody nearly abolished the staining (insert). (**B**) GCs, corpus luteum and surface epithelium show staining for AChE. (**C**) The staining nearly disappeared in the preadsorption control.

To assess the ovarian distribution of AChE we performed immunohistochemistry in ovarian tissue slices. We found a preferential localization of AChE associated with blood vessels and specifically with GCs of different classes of follicles (Fig. 2A). The enzyme was also detected in the corpus luteum (CL; Fig. 2B,C) and in the surface epithelium of the ovary. Preadsorption controls confirmed the specificity of the results.

### Participation of AChE in the *in vivo* function of the ovary

**Figure 3:**
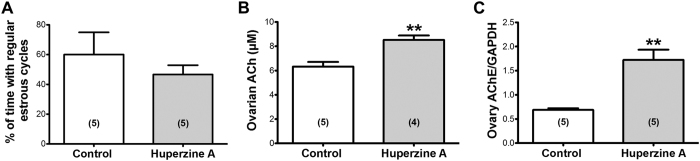
Effects of HupA on estrous cyclicity, ACh levels and AChE expression. (**A**) No significant change in estrous cyclicity of the animals was observed. (**B**) Ovarian ACh levels were increased by HupA treatment. (**C**) Increased AChE protein levels in the HupA group determined by western blot analysis. All values correspond to n = 5 control animals and n = 5 or n = 4 animals treated with HupA (2 μM) for 28 days. Results are the mean + S.E.M. (*P < 0.05; **P < 0.01).

We examined the consequences of *in vivo* pharmacological blockage of AChE by HupA locally applied to the left ovary of hemiovariectomized rats. Saline was used in the control animals. In the presence of HupA there was no modification of estrous cyclicity (Fig. 3A).

Ovarian ACh concentrations were determined and as seen in Fig. 3B, a significant increase in ovarian ACh was found in the HupA group. This indicates that the delivered HupA was effective in blocking the ACh metabolizing AChE.

To examine whether AChE is a factor, which locally regulates the autonomic induced changes in ovary function, we measured the protein expression of AChE in the presence of HupA (Fig. 3C). The inhibition of AChE for 4 weeks leads to a twofold increase of AChE mRNA levels.

### Effects of modifying ACh bioavailability on ovarian follicular development

**Figure 4:**
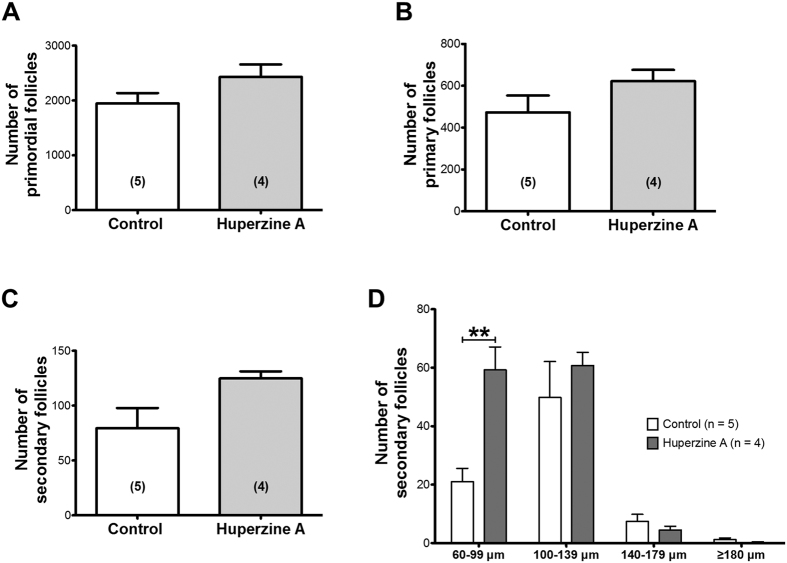
Effects of HupA treatment on the ovarian preantral follicular development in the rat. (**A**) Number of primordial follicles in the rat ovary shows no significant change after HupA treatment. (**B**) No change of the primary follicle count was observed in HupA group compared to control. (**C**) HupA treatment of the rat ovary trends to result in an increased occurrence of secondary follicles. (**D**) Values for the number of secondary follicles corresponding to the mean diameter revealed an increase of small secondary follicles (60–99 μm) after HupA exposure. All values are shown as mean + S.E.M. of n = 5 (control) and n = 4 (HupA) individual rats (**P < 0.01).

**Figure 5:**
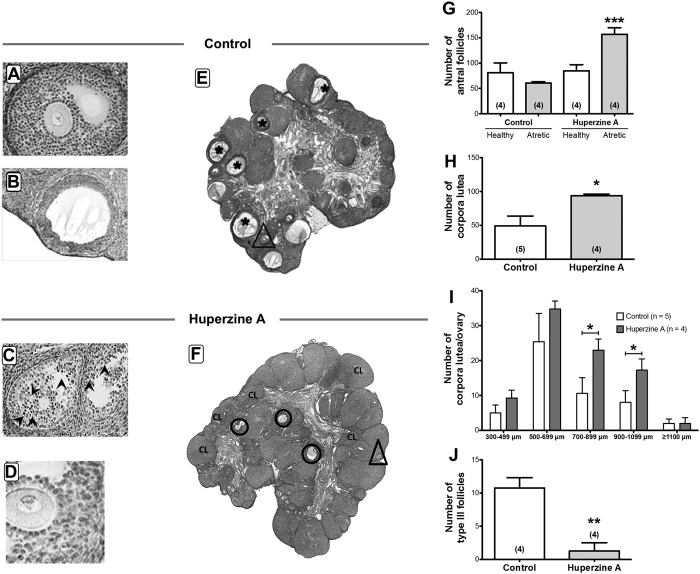
Effects of HupA on the ovarian follicular development in the rat. (**A**) Representative picture of a healthy antral follicle of a control ovary. (**B**) Example for a precystic follicle appearing in the control group. (**C**) Example for an atretic follicle of a HupA treated ovary. (**D**) Example for a healthy follicle of a HupA treated ovary. (**E**,**F**) Sections of a control (**E**) and a HupA treated (**F**) ovary are depicted to highlight increased occurrence of CL and atresia of antral follicles (ellipses in F and magnification in C, in which arrowheads show atretic cells, in comparison to a healthy antral follicle (**B**) after HupA exposure. Asterisks indicate precystic follicles and triangles show healthy antral follicles. (**G**) Quantification of the number of antral follicles (healthy and atretic) showed an increase of atretic antral follicles in the HupA group. (**H**) CL number is significantly increased after HupA treatment. (**I**) Values for the number of CL corresponding to the mean diameter. An increased number of CL with 700–899 μm and 900–1099 μm diameter after HupA exposure was observed. (**J**) Decrease in number of precystic type III follicles after HupA treatment. All values are shown as mean + S.E.M. of n = 4 or n = 5 individual control or HupA-exposed rats (*P < 0.05; **P < 0.01; ***P < 0.001).

Figure 4 shows the effects of 4 weeks of exposure to HupA on follicular development. We quantified the primary and secondary preantral follicle population using serial sectioning. No changes were found in the number of primordial and primary follicles (Fig. 4A,B), however we observed a tendency of an increased number of preantral secondary follicles (Fig. 4C). HupA caused a significant accumulation of the smaller sized preantral secondary follicles (Fig. 4D), suggesting that the effect of HupA is focused on the promotion of growth of primary follicles. More effects of HupA were observed by analyzing ovarian sections (Fig. 5A–H). Although no important changes occurred in the number of healthy antral follicles, there was an increase in the frequency of atresia of the antral follicles (Fig. 5I). When we analyzed the number of CLs (Fig. 5J), we also found an increase in the number of total CLs that was especially significant in the CLs in the higher range of size (Fig. 5K). Furthermore we observed a non-expected decrease in the number of precystic type III follicles (Fig. 5L).

### Effects of HupA on fertility of the rat

**Figure 6:**
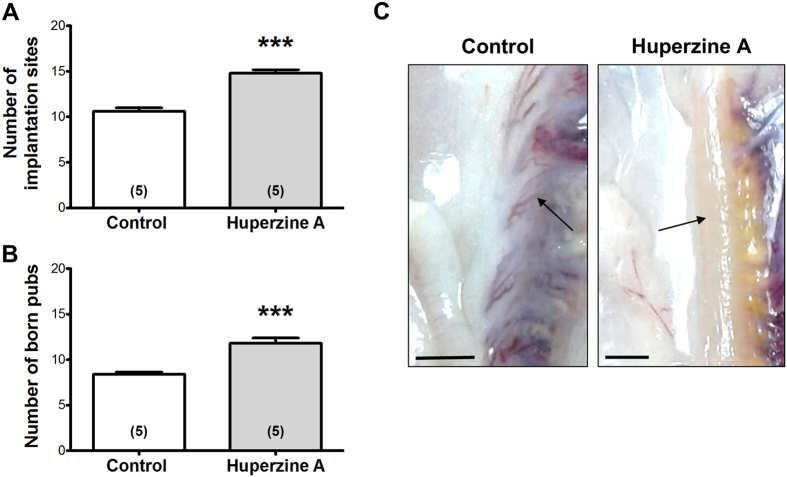
Effects of intraovarian HupA on fertility of the rat. (**A**) Number of implantation sites per rat found either in the uteri of control or HupA treated rats. HupA treatment leads to a significant increase of implantation sites. (**B**) Increased number of born pubs per rat in the HupA group compared to control. All values are shown as mean + S.E.M. of n = 5 individual control or n = 5 HupA-exposed rats (***P < 0.001). (**C**) Pictures of uteri of control and HupA-treated rats in which the implantation sites (arrows) remain after delivery. Bars indicate 1 cm.

Figure 6 shows the number of pups born after HupA or saline treatment of the rats. There is an increase in both the number of pups born (Fig. 6B) and also in the number of uterine implantation sites (Fig. 6A,C). There was no change in the length of pregnancy and also in the weight or sex of the newborn (not shown).

## Discussion

This study provides evidence that ACh is involved in the regulation of ovarian functions *in vivo* in the rat. This conclusion is based on the consequences of *in vivo* inhibition of ovarian AChE by HupA, which resulted in elevated ovarian ACh, enhanced follicular development of small follicles, reduced formation of cysts, enhanced ovulation and increased numbers of pups. These results reveal for the first time the importance of local, ovarian ACh in the control of follicular development and suggest that pharmacological manipulation of AChE may be a novel way to interfere with ovarian functions.

AChE, identified at mRNA and protein levels, was localized to endocrine and vascular compartments of the rodent ovary. The enzyme was principally associated with blood vessels, follicular GCs and the CL, suggesting that during adulthood AChE is principally associated with blood vessels and the rapidly changing endocrine compartments of the ovary. The results obtained in rat are, in general, in line with the one in human and monkey ovary, in which AChE was recently found specifically in the GCs of ovarian follicles^9^. While a cholinergic innervation of the ovary is matter of dispute and cannot be completely excluded, the GCs of the follicles certainly are completely devoid of nerve fibers^7^,^13^,^14^. Hence the role of AChE in the follicle is related to the termination of the actions of ACh produced by GCs. These cells also express muscarinic receptors^13^,^14^. AChE thus acts as a local regulator of the availability of ACh in the follicular compartment. This has also been suggested by a study of the bovine CL^8^.

In the present work we used a previously developed technique for *in vivo* intrabursal delivery of drugs to the ovary^15^,^16^. The local administration of HupA blocked the activity of AChE and thus increased the availability of ACh in the ovary, as shown by the increased concentration of ovarian ACh. The measured small but significant increase in overall ovarian ACh concentration might have been more pronounced if we have added a blocker of BuChE, which was also detected and in substantially higher amounts than AChE.

Our observation that the local increase in ACh did not modify estrous cycling activity of the rats implies that HupA treatment did not affect the hypothalamic pituitary axis and thereby the ovary. We also obtained some insights into estradiol and progesterone levels in HupA-treated rats and controls (results not shown). We measured both sex steroid hormones when the animals were euthanized and presented diestrus. We did not observe significant differences, a result that is in agreement with the undisrupted estrous cyclicity and fertility. Taken together the results strongly suggest that the hypothalamic pituitary ovarian axis is unaffected.

Thus HupA treatment appears to have a restricted effect on the ovary only. The blockage of AChE with HupA, not only increased the ACh present in the ovary, but also the protein levels of ovarian AChE. This may be a compensatory mechanism in an attempt to retain tissue homeostasis of ACh, or could be due to other reasons, including a non-fully functional enzyme. A dissociation between the activity and amount of enzyme protein could be the result of impaired processing at the endoplasmic reticulum as has been demonstrated to occur to other neuronal markers^16^. A series of biochemical experiments to study the processing of the enzyme will be required to clarify this possibility.

Importantly, the inhibition of AChE by HupA for 4 weeks presumably via the associated elevations of intraovarian ACh, strongly modified follicular development. Initial follicular growth was not significantly changed, evidenced by comparable numbers of primordial and primary follicles. However we found an increased number of preantral secondary follicles. Specifically, an accumulation of small preantral secondary follicles became evident, suggesting that the effect of HupA, i.e. the action of ACh, is focused on the promotion of the growth of primary follicles. The fact that ACh increases the proliferation of GCs via muscarinic receptors was recently confirmed by using human IVF-derived human GCs^9^. Since cell proliferation is the driving force of follicular growth this mechanism may be responsible for the observed promotion of follicular development. If so, our results also suggest that the effect of ACh is targeted to specifically increase the transition from preantral primary/secondary follicles to antral follicles.

Interestingly there was also an increase in the rate of atresia of antral follicles upon HupA treatment and we observed more CL. The reason for these results remains to be fully elucidated. Atresia occurs constantly in the ovary and it is unlikely that this process may have an overall negative effect on follicular growth. Yet, the growth of antral follicles is thought to depend on the hormone FSH. ACh may thus drive the initial development of follicles to the antral stage, but many of those follicles may then undergo atresia likely due to a lack of support by FSH. However, the increase in the number of medium-sized CL indicates that in addition to the observed enhanced growth of small follicles and the stimulation of atresia, some follicles have grown further and reached the ovulatory stage. In support for this, we found that after mating more implantations occurred in HupA-treated rats than in saline controls and importantly, more pups were born after HupA treatment. There was a good correlation between the number of pups born and the number of implantation sites (an average of 1–2 implantation sites difference compared with the number of pups born), which allow us to conclude that pregnancy was not affected by the treatment.

In order to eliminate a possible effect of the contralateral ovary we used hemiovariectomized rats for this study. We observed that hemiovariectomized rats spontaneously developed precystic follicles. This is uncommon in normal, 2–3 month old rats and strongly suggests that long-term hemiovariectomy predisposes the animal to develop follicular cysts. A type III follicle represents a precystic follicle, as previously described^15^,^17^,^18^,^19^. We found that 80% (4 of 5) of rats presented type III precystic follicles^15^. Interestingly, only 25% (1 of 4) of the hemiovariectomized rats exposed to HupA developed type III follicles, and 50% of the rats did not present cystic structures at all, suggesting that HupA/increased ACh level have a protective effect and foster a healthy ovulatory process. This is in line with and related to the observed promotion of follicular growth and of ovulation.

There are a number of uncertainties and open questions. While HupA increased ovarian ACh and thus did diffuse into the ovarian tissue, we do not know whether HupA delivered to the bursa may have reached all types of follicles in the ovary equally and to what extend other ovarian cellular sites, which express AChE, are targeted. Due to the architecture of the ovary, small follicular stages are typically located in the ovarian cortex and presumably are reached first. Therefore they could be affected more than larger follicles, which have a different position within the ovary and are further away from the cortex of the gonad. Also, it remains to be shown whether other blockers of AChE are able to induce the same changes in order to exclude possible non-specific modes of actions of HupA. Finally, although large blood vessels are mainly in the medulla of the ovary, it is possible that ovarian vasculature and changes in blood flow also may have contributed to the observed changes. Nevertheless, the observed changes in HupA-treated ovaries in combination with the production of ACh by GCs and the documented trophic action of ACh on GCs, are in complete agreement with a specific mode of action of HupA to imbalance the ovarian cholinergic system.

The changes in follicular development and in ovulation promoted by ACh are in striking contrast to the actions of NE, the neurotransmitter of the sympathetic innervation of the ovary. We and others^20^,^21^,^22^ have demonstrated that increases in the sympathetic tone and in ovarian NE are directly related to a deranged follicular development, and are associated with a polycystic ovary syndrome, the most frequent ovarian pathology during reproductive years in women. Our present results strongly suggest that ACh acts as a trophic compound for the ovarian follicular development and ovulation and implies that we need to develop experimental protocols to manage both systems.

## Material and Methods

### Animals

Adult female Sprague Dawley rats between 250–300 g from our facilities were used. Rats were maintained with food and water ad libitum and a 12:12 hours day:night cycle. The experiments with HupA were designed to achieve an effective local intraovarian concentration of HupA, a specific pharmacological blocker of AChE^12^. The constant delivery of the drug was achieved by means of an Alzet osmotic minipump (model 2004, Alza Corp., Palo Alto, CA, USA) filled with a 10 μM HupA solution or with saline, in the case of sham operated control. The implantation method has been used previously in our laboratory^15^,^23^. In order to eliminate the contribution of the contralateral ovary to the changes in reproductive function produced by HupA, we used hemiovariectomized rats (removal of the right ovary), which were divided into two main groups: sham control rats (n = 25) implanted with the minipump filled with saline. In the second group (n = 25) the minipump was filled with 10 μM HupA solution. Both control and HupA-treated rats were examined by daily vaginal smears to verify estrous cycle regularity. Results are presented as the percentage of regular cycling activity considering the following stage of the estrous cycle: proestrus (P), estrus (E), diestrus (D). Control rats presented a regular 4-days estrual activity. Rats were euthanized by decapitation at 11:00 h of the diestrus phase of the estrous cycle. To study fertility, 5 of the HupA-treated rats and 5 control rats were mated with fertile male the night of proestrus. Either control or HupA treated rats were checked for the presence of sperm forming the vaginal plug the other day. Rats with Sperm positive rats were assigned with pregnancy day-1. During delivery, born pups were counted and maintained with the mother during 3 days. At day 3 mothers were euthanized to verify the implantation sites in the uterus and pups were derived to other lactating mother. Ovaries were used for histology, biochemical measurements, morphometry and ACh determination and each experimental group contained five replicates. All experimental procedures were approved by the Bioethics Committee of the Faculty of Chemistry and Pharmaceutical Sciences at the Universidad de Chile and complied with national guidelines (CONICYT Guide for the Care and Use of Laboratory Animals). All efforts were made to minimize the number of animals used and their suffering.

### Isolation of granulosa and residual ovary cells fraction

Granulosa cells were collected as described previously^24^,^25^. Briefly, ovaries were punctured with a needle, and the cell suspension was carefully expressed into Krebs bicarbonate buffer. The cells were transferred to a 1.5 ml plastic tube, pelleted by centrifugation at 3000 × g, and washed three times with Krebs bicarbonate buffer. With this method we have previously found that more than 90% of the mRNA for FSH receptor is located in the granulosa cell fraction^24^. Both the suspension of granulosa cells and the rest of the ovary (residual ovary containing theca-interstitial cells, blood vessels, nerve terminals, etc.) were used for extraction of total RNA to do the RT-PCR or qPCR.

### AChE expression in the rat ovary

To examine BuChE, AChE and isoforms, we used RT-PCR and qPCR with mRNA obtained from the complete rat ovary or the granulosa theca-interstitial cell fractions. RNA extraction from 10 mg of tissue or the isolated fractions of cells was done using the Total RNA E.Z.N.A Kit I (Omega Bio-Tek, Norcross, GA, USA) according to the supplier’s instructions. We verified the integrity of the RNA with an agarose gel electrophoresis and the total amount of RNA was quantified with the Kit Quant-IT RNA Br (Molecular Probes, Carlsbad, CA, USA). A total of 5 μg of total RNA was incubated with reverse transcriptase SuperScripT II, Invitrogen, Carlsbad, CA, USA) in a final volume of 20 μl according to Dorfman *et al*.^5^. For qPCR analysis we used IQ5 thermocycler (Bio-Rad, Hercules, CA, USA) with Brilliant II SYBR^®^Green QPCR Master Mix (Agilent Technologies, Santa Clara, CA, USA) using 2 μl of a 1/10 dilution of the cDNA previously obtained. The primers used to detect BuChE, were obtained from Mis *et al*.^26^. The sense primer was 5′AGAATGGATGGGAGTAATGCATGG3′, the antisense primer was 5′GATGGAATCCTGCCTTCCACTCTTGC3′. To detect AChE the sense primer 5′CCCATGGCTATGAAATCGAG3′ and the antisense primer 5′TTCAGGCTCACG TATTGCTC3′ were chosen based in the sequence information given NM_172009.1. In this case we sequenced to verify authenticity. To detect the AChE isoforms we used the common sense primer described by Sifringer *et al*.^27^ 5′CAGCAATACGTGAGCCTG3′. To detect the S isoform we chose the antisense primer 5′GGTCGAACTGGTTCTTCCA 3′^27^. For the H isoform we used the antisense primer 5′TTAGAGCCACCGAAGCCCGG3′ and for the R isoform we used the antisense primer 5′CTTCCAACCCTTGCCGCC3′, both obtained from Legay *et al*.^28^. For qPCR determination of each transcript we used a 0.16 μM solution of each primer and the standard protocol of the supplier. Polymerase was activated at 95 °C for 10 minutes, followed by 40 cycles of alignment at 58 °C elongation at 72 °C. To quantitate we used 18S transcript as a constitutive gene.

### Western blot analysis

For western blot the ovary was homogenized in 10 volumes of RIPA buffer (1% NP40, 0.5% sodium deoxycholate, 0.1% SDS in PBS; just before use add 10 μl of the following mixture (10 mg/ml stock solution of PMSF; Aprotinin and sodium ortovanadate ) in the presence of Complete Mini EDTA-Free Protease Inhibitor Cocktail (Roche, Basel, Switzerland). Proteins extracted were quantified with Lowry method^29^ and 30 μg were run on 10% polyacrylamide gel. Proteins were transferred to nitrocellulose and incubated with an antibody that recognizes all the isoforms of AChE (E-19, Santa Cruz Biotechnology, Dallas, TX, USA). As a positive control we used a total extract from rat brain and the negative control was done with the preadsorbed antibody with the immunogenic peptide (E-19p, Santa Cruz Biotechnology).

### Immunohistochemistry

The method was described previously^9^. In brief, ovaries were fixed in Bouin’s fluid, embedded in paraffin and cut at 6 μm each. Antigen recovery was done by microwave heating at 800 W for 10 minutes in buffer 10 mM citrate pH 6.0, washed 3 times with PBS. Samples were incubated for 30 minutes in 3% hydrogen peroxide with 10% methanol to block endogenous peroxidases. The AChE the polyclonal antibody E-19 from Santa Cruz Biotechnology recognizes all AChE isoforms and was used. Incubation was done overnight with 1:50 antibody solution in PBS in the presence of 5% normal donkey serum. On the second day the tissue was washed in PBS and incubated with the secondary biotinylated antibody (1:500) for 2 h. To detect the signal we used Vectastain ABC kit (VectorLabs, Burlingame, CA, USA) according to manufacturer instructions. Preadsorption was done as described for Western blotting.

### Determination of ovarian endogenous ACh levels

To quantify the ACh concentration in the ovary, a complete ovary was homogenized in 10 volumes of PBS in ice and the neurotransmitter determination was done using the Amplex Red Acetylcholine Assay (Invitrogen) as described previously^30^.

### Acetylcholinesterase activity in the rat ovary.

The ovaries of 5 rats were obtained, weighted and suspended in 10 volumes of ice-cold phosphate-buffered solution (pH 7.0). The ovaries were homogenized in a glass-glass homogenizer and centrifuged at 10000 × g for 10 minutes. 15 μl of the supernatant was used to determine AChE by the method of Ellman, as described by Blohberger *et al*.^9^.

### Morphometric analysis

Ovaries previously fixed were embedded in paraffin, cut into 6 μm sections, and stained with hematoxylin and eosin. Morphometric analyses of whole ovaries were done as previously described^15^,^31^ using n = 4 ovaries of HupA and 5 saline controls. All follicular structures were followed through all slices and were counted when they reached the largest diameter. Primordial follicles were those with one oocyte surrounded by flattened granulosa cells; primary follicles were counted as follicles exhibiting one layer of cubical granulosa cells; secondary follicles had no antral cavity but two or more layers of granulosa cells; atretic follicles had more than 5% of cells with pyknotic nuclei in the largest cross-section and exhibited shrinkage and an occasional breakdown of the germinal vesicle; antral follicles were those with more than 3 healthy granulosa cell layers, the antrum and with a clearly visible nucleus of the oocyte; type III follicles were large follicles containing four or five plicated layers of small, densely packed granulosa cells surrounding a very large antrum with an apparently normal thecal compartment; precystic follicles do not present the oocyte but they still have many layers of granulosa cells; finally, cystic follicles were devoid of oocytes and displayed a large antral cavity, a well-defined thecal cell layer, and a thin (mostly monolayer) granulosa cell compartment containing apparently healthy cells^15^. All abnormal follicular structures were grouped as cystic structures.

### Statistical analysis

The data are expressed as the mean + S.E.M. To determine statistical differences between 2 groups, we used two-tailed Student’s *t*-test. To analyze differences between several groups we used one-way analysis of variance (ANOVA) followed by a Newman-Keuls post-test (see results shown in Fig. 1B). The results shown in Fig. 1D were analyzed using column statistics (Prism GraphPad, La Jolla, Ca, USA).

## Additional Information

**How to cite this article**: Urra, J. *et al*. In vivo blockade of acetylcholinesterase increases intraovarian acetylcholine and enhances follicular development and fertility in the rat. *Sci. Rep.* **6**, 30129; doi: 10.1038/srep30129 (2016).
